# Social isolation and loneliness among older adults living in rural areas during the COVID-19 pandemic: a scoping review

**DOI:** 10.1186/s12877-023-04196-3

**Published:** 2023-08-23

**Authors:** John Pickering, Andrew V. Wister, Eireann O’Dea, Habib Chaudhury

**Affiliations:** 1 Gerontology Research Centre, Simon Fraser University, 2800-515 Hastings Street Vancouver, Burnaby, BC V6B 5K3 Canada; 2 Department of Gerontology, Simon Fraser University, British Columbia, Canada

**Keywords:** Rural, COVID-19, Social isolation, Loneliness, Older adults

## Abstract

**Background:**

The causes and consequences of social isolation and loneliness of older people living in rural contexts during the COVID-19 pandemic were systematically reviewed to describe patterns, causes and consequences.

**Methods:**

Using the Arksey and O’Malley (2005) scoping review method, searches were conducted between March and December 2022, 1013 articles were screened and 29 were identified for data extraction.

**Results:**

Findings were summarized using thematic analysis separated into four major themes: prevalence of social isolation and loneliness; rural-only research; comparative urban-rural research; and technological and other interventions. Core factors for each of these themes describe the experiences of older people during the COVID-19 pandemic and related lockdowns. We observed that there are interrelationships and some contradictory findings among the themes.

**Conclusions:**

Social isolation and loneliness are associated with a wide variety of health problems and challenges, highlighting the need for further research. This scoping review systematically identified several important insights into existing knowledge from the experiences of older people living in rural areas during the COVID-19 pandemic, while pointing to pressing knowledge and policy gaps that can be addressed in future research.

## Background

First identified in late 2019, the coronavirus disease (COVID-19) has resulted in a global pandemic, particularly affecting the lives of older adults owing to their higher likelihood of having pre-existing conditions or multimorbidity, and of being immune compromised [1–4]. It is a highly contagious viral disease that has been associated with a spectrum of deleterious health effects, ranging from flu-like symptoms to death. Older adults, particularly those in institutional settings such as long-term care facilities (LTC), have been the most affected by the serious complications of COVID-19 infection and have accounted for the highest percentage of deaths due to the virus [4, 5]. However, those living in the community have also experienced a higher risk of negative physical and mental health consequences [6, 7]. The high-risk of infection and its negative health consequences among older adults have prompted the use of the new term “geropandemic” among researchers [e.g., 8, 9]. Furthermore, media reports have heightened the awareness of these high-risk populations, which have in turn intensified feelings of vulnerability, fear, stress and anxiety. Public health policies to control the spread of the disease in many countries included “lockdowns,” where they restricted work environments or travel in and out of the country, closed schools, and limited overall mobility. These restrictive measures were deemed necessary to slow the COVID-19 transmission rates and to safeguard health institutions and resources from becoming overwhelmed by an unprecedented number of patients [10]. The subsequent isolation related to the lockdowns affected lives across geographic spaces at the global, regional, national and local levels [9]. The pandemic experiences and responses, including the effects of social isolation and loneliness, have been highly diverse depending on the level of urbanity or rurality level of environments [e.g., 11, 12]. These knowledge gaps have led to calls for reviews examining the effects of the pandemic on older adults living in rural environments [e.g., 13]. The present scoping review responded to these calls and aimed to analyze research studies that specifically examined the effects of social isolation and loneliness among older adults living in rural or remote communities.

### Social isolation and loneliness

Social isolation and loneliness (Si/L) are increasingly deemed to be public health challenges with unique ageing associations. Social isolation has been typically defined, “as a lack in quantity and quality of social contacts” and “involves few social contacts and few social roles, as well as the absence of mutually rewarding relationships” [14, p.1]. Loneliness is usually defined as “a distressing feeling that accompanies the perception that one’s social needs are not being met by the quantity or especially the quality of one’s social relationships” [15, p.218]. In this paper, social isolation is defined as a separation from social connections, such as friends, family, acquaintances and loved ones. Loneliness, on the other hand, is defined as the subjective experience, often due to social isolation, but not necessarily. The terms can be mutually exclusive because it is possible to be separated from social contacts and not feel lonely, or to be in close contact with friends and family, but still experience feelings of loneliness [16]. The geography of the rural environment further complicates this separation of Si/L because residents are often simultaneously cast as at-risk in terms of health outcomes [e.g., 17–19], yet benefitting from higher levels of social cohesion activities (e.g., community participation, volunteering, etc.) as compared to urban areas [e.g., 20–23]. These nuanced understandings of one’s environment are important in determining how these experiences are recorded and measured by researchers. Social isolation is measured using quantitative measures of physical separation from an individual or network perspective. The subjective experience of loneliness is measured or understood based on quantitative scales or qualitative methods to capture individuals’ lived experiences. We do not review different conceptualizations and/or measurements of these concepts, but rather, focus on their patterns across rural-urban environments among older adults during the COVID-19 pandemic, given that the nexus of these factors is deemed to be relevant [24].

### Research challenges studying rural environments, isolation and COVID-19

An important challenge for researchers conducting studies on rural environments is related to the definition of *rural*. Rurality is often given a residual definition, which defines it as what it is not (i.e., urban space), as opposed to what it is. The definition of urban areas often becomes the primary tool to determine what is and is not rural, but this differs greatly across studies and geographical context. For example, Canada defines urban centres as having a population of > 1000 and a population density of > 400 people per square kilometer [25]. Lower middle-income countries (LMICs) in the global periphery often lack clear, official definitions. Nigeria, for example, defines rural places as having a population < 20,000 and with a primary economic focus on agriculture, according to the United Nations Department of Economic and Social Affairs [26]. Our interest is centered on older adults who live in areas locally defined as “rural,” rather than attempt to impose a particular definition of “rurality” on the included studies. Despite the abundance of literature on ageing and rural environments, few studies have focused on this in the context of the COVID-19 pandemic.

In many ways, social isolation is a *de facto* element of living in a rural environment as compared to an urban one, due to lower population density and physical distance between residences. Research has been equivocal in terms of levels and outcomes of social isolation and loneliness among older adults based on levels of rurality /urbanity of the environment. Henning-Smith and colleagues (2019) reported these equivocal findings based on a study of nearly 2,500 older adults living in rural, small towns and metropolitan urban centres [27]. The findings suggest that urban centre residents were more likely to feel socially isolated and lonely. However, significant differences existed among race and ethnicity divisions, and rural residents were found to be more at-risk for loneliness than their urban dwelling peers. Similar differences were found in differing measures of social contact, such as access to social services and social capital. For instance, research has established that older adults in rural environments are disadvantaged with respect to access to community and health services [e.g., 17–19, 28]. This association points to the issue of blurring between social and physical isolation. Yet, other research indicates that rural older adults have higher levels of social capital (i.e., stronger community connectedness), resulting in rich and more satisfying social engagement and support from neighbours and the broader community [e.g., 20–22]. Additionally, the role of technologies during Covid-19 to reduce social isolation and loneliness among older adults has been supported, although a focus on rural environments remains under-researched [29]. The recent COVID-19 pandemic has created increases in Si/L for most older adults due to public health behaviours and policies. Whether and to what degree rural older adults have experienced Si/L differentially than their counterparts in more urban environments is largely unknown.

### Rationale for study

Despite the extensive literature addressing causes and consequences of Si/L among older adults spanning decades of research [see 30–34], systematic reviews of this knowledge in relation to the lived experiences in rural settings are few. Furthermore, a focus on the existing research conducted during the COVID-19 global pandemic represents a novel study and an organic experiment to study rural experiences of older adults under adverse conditions. Hence, we propose a scoping review to ask the following overarching question: what is known about the social isolation and loneliness of older adults living in rural settings during the COVID-19 pandemic? Our initial research questions include: (A) how did COVID-19 affect the prevalence of Si/L among older adults? (B) what factors contributed to Si/L among older adults during the pandemic? (C) how did Si/L affect the lives of older people during COVID-19; and (D) how were technological interventions employed to address Si/L among older adults during the pandemic? This review aims to synthesize the factors of the Si/L of older adults living in rural environments during the COVID-19 pandemic, describe the state of the existing literature, and identify key knowledge gaps systematically to facilitate subsequent research for further policy and program development.

## Methods

Scoping reviews are a form of knowledge synthesis that aim to identify the key themes or concepts informing a particular area of research and summarize the main types of evidence and sources available based on a multi-step iterative process [35–37]. Scoping reviews are particularly useful when the literature on a topic has employed a range of data collection methods and/or analysis techniques, there is a lack of previous knowledge syntheses on the topic, and/or the project does not require a quality assessment of the included studies [35]. Our study topic meets all three criteria, which is why we have elected to employ the scoping review method. Our approach involves several procedural steps and the scoping review began in March 2022. The preliminary list of sources for inclusion was identified in December 2022, with the full article review having been completed in January 2023. We detail the procedural steps, derived using the scoping review method characterized by Arksey and O’Malley (2005), below [35].

### Step 1 - identifying the question and relevant literature

We began by searching for existing reviews to determine the general knowledge and synthesis gaps relevant to our interest on how COVID-19 has affected older adults’ social lives in rural areas. *Since n*o existing knowledge syntheses were found at the time of our scoping review, we refined our synthesis question to fill this knowledge gap. Following this, we devised a library data base search strategy to determine specific keywords specific to gerontological inquiry. In addition, we hand searched relevant titles and abstracts of recent studies to supplement the data search. Table 1 details our search strategy using identified keywords, which focused on four categories. After performing preliminary search attempts it was determined that some terms were responsible for erroneous returned results. The table was refined by utilising Boolean terms to better manage the returned search results employing an iterative process. For example, terms that further focused the search on the COVID-19 pandemic and those which limited or eliminated articles focusing on institutional care, were also identified and included at this stage. This process also helped to maintain a focus on rural areas due to the lack of institutional and long-term care provision in rural places.

**Table 1:** Scoping review keyword search strategy

Focus	What/Where	Who	Why
“Loneliness”	Rural	“Older adults”	COVID*
“Social isolation”	Remote	“Elderly”	n-Coronavirus
	Countryside	“Seniors”	Pandemic
	Village		Restriction

* boolean terms added to all searches: (-“nursing home”), (-“long-term care), (-“senior center”), (-“HIV”), and (-“AIDS”)

### Step 2 – searching the literature

A search strategy was used that included English-language, peer-reviewed literature published in scholarly journals, and our search strategy was developed to specifically target journals focusing on older adults and gerontology. These inclusion criteria were chosen for specific reasons, including: a lack of language ability outside of English on the review team; peer-reviewed articles published in quality journals maintains a high level of research incorporated into the review; and older adults were a criterion since a gerontological focus directed this study. Relevant combinations of keyword terms from Table 1 were searched in eight academic databases, including Google Scholar (Table 2). Boolean operators were used to maximize the combinations and permutations of the terms, and various combinations yielded marginally different results. Using an iterative process, additional keywords were added and Boolean terms and/or removed keywords when the returned results were irrelevant and/or unproductive.

**Table 2:** **Academic Databases Searched, IRM Scoping Review**

Database	Temporal Period Covered (dd/mm/yyyy)
Ageline	01/01/2020–16/12/2022
Biomed Central	01/01/2020–02/11/2022
Global Health	01/01/2020–10/09/2022
Google Scholar	01/01/2020–16/12/2022
PsycINFO	01/01/2020–02/11/2022
PubMed	01/01/2020–16/12/2022
Sociological Abstracts	01/01/2020–10/09/2022
Web of Science	01/01/2020–24/10/2022

Building on our consultations with the reference librarian, we used the gerontology-specific databases to conduct our search. We targeted Google Scholar for our preliminary database search used to identify exclusion terms. Certain terms, including nursing home and pandemic, consistently yielded unwanted results and were removed from subsequent searches by adding (-“nursing home”), (-“long-term care”), (-“senior center”), (-“HIV) and (-“AIDS”). Our preliminary Google Scholar searches indicated that this strategy would be unlikely to exclude potentially usable articles. Identical search queries were conducted in the database searches and were further focused using options within the ‘who’ category, as necessary. Some of the terms included within the “who” category are considered to be examples of ageist language (i.e., seniors); however, we decided to include these terms to maximize our search results. Search results were then organized and stored using Zotero reference management software and inputted into the Covidence review managing software and a shared Google Sheet.

### Step 3 – charting the data

We used the Zotero software to remove duplicate sources. Our first step in data charting was to independently review the titles and abstracts to identify articles to be reviewed in full. Subsequently, the authors selected those that should be read in full for potential inclusion in the scoping review. Eliminated sources during the title/abstract review stage included (1) articles that lack focus on the synthesis question (e.g., social isolation, loneliness, COVID-19, and rural location), (2) materials that are not peer-reviewed published articles, and/or (3) sources not written in English.

Articles identified for full review were gathered through institutional journal subscriptions or inter-library loan. Two team members were assigned to read each article to determine if it should be included in the review. Authors independently reviewed articles remotely and recorded notes on the source and its viability for inclusion. The lead author reviewed the recommendations to reach consensus on the inclusion and exclusion criteria and the scope of the extracted data. There were few initial disagreements related to inclusion or exclusion of the sources among the team members. In the few instances where consensus was not achieved between two readers, inclusion or exclusion was determined by a third reader to make the final determination. The main reasons for exclusion at this stage were a lack of clear focus on social and isolation and/or loneliness during the COVID-19 pandemic, a lack of focus on older adults, or a distinction between rural- and urban-based older adults. During this step, we also hand-searched the reference lists of included articles to identify other sources that could undergo a second round of title and abstract review.

### Step 4 - collating, summarizing and reporting the results

The review process was charted on the Covidence review managing software, and a secure, online spreadsheet editable by all team members and based on the authors’ previous experiences with scoping reviews [e.g., 29, 38]. We independently recorded bibliographic details of each study and extracted data for the reviewed articles. At the completion of the review phase, the extracted data were reviewed independently by each team member to identify themes and organise the findings relevant to the synthesis question. The lead author then inputted the included articles into QSR NVivo to code the data points and assist in the definition and identification of relevant themes. We then held remote correspondence to identify key themes and define their scope and scale. The following section details the four synthesis themes identified.

## Findings

**Fig. 1:**
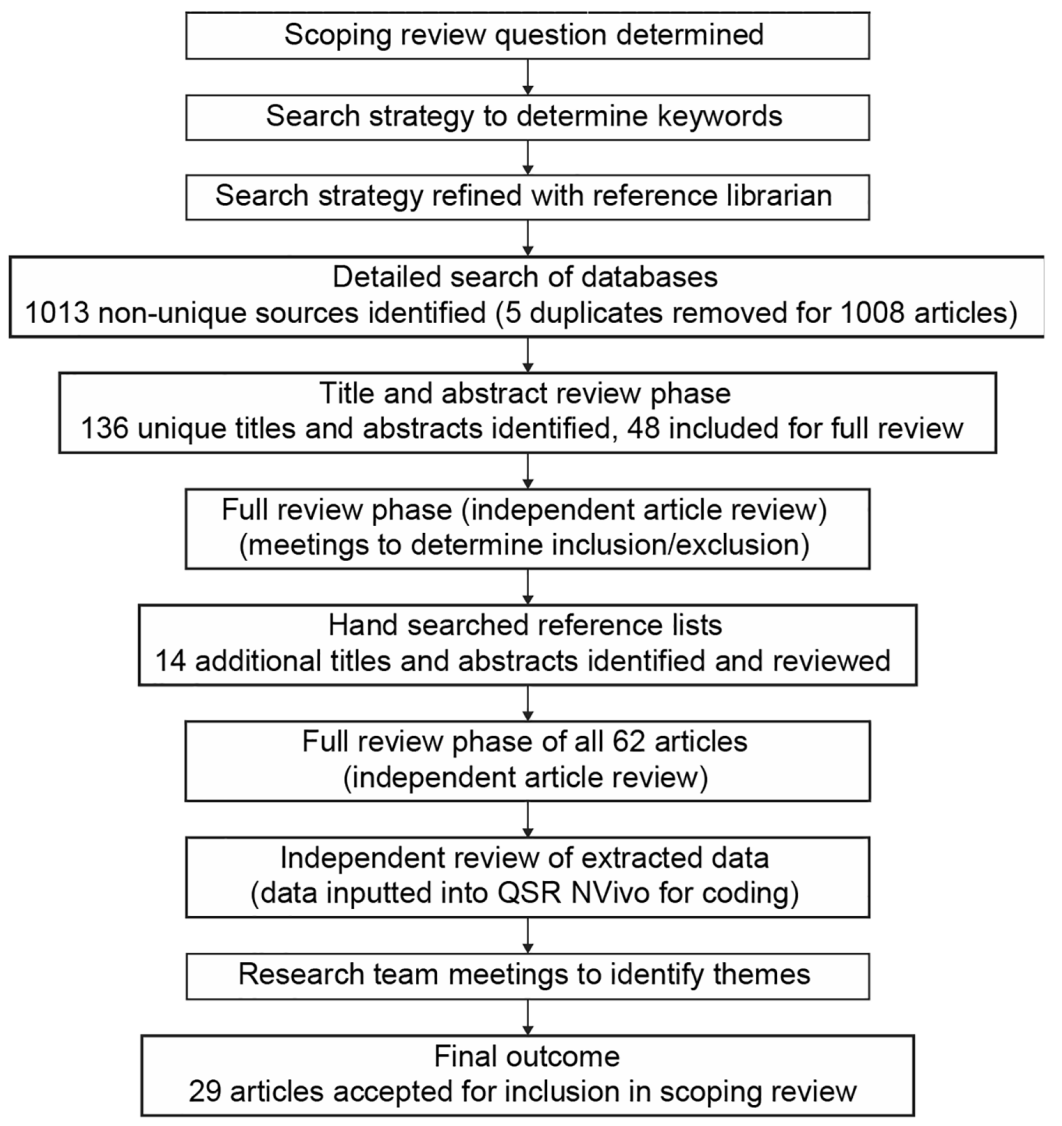
Scoping review process and results

Figure 1 provides a summary of our scoping review search process and outcomes. Twenty-nine articles met the study criteria and were included in the review. Employing an inductive thematic analysis approach [39, 40], we followed Braun and Clarke’s (2006) steps for thematic analysis: (i) familiarization, (ii) generation of codes, (iii) search and review themes, and (iv) theme definition. From the included articles we organized the review results according to the following four main crosscutting themes: (1) Prevalence of social isolation and loneliness, (2) Rural-only research (all participants lived in a rural environment with no comparison to non-rural group), (3) Urban-Rural comparative research, and (4) Technological and other interventions (see Table 3 with this organisation, and Table 4 for the methodological breakdown of the included studies). A further summary of all articles included for review can be found in Table 5. We further separate these results into positive or negative findings relating to each theme. While the prevalence and technological application themes align with the questions 1 & 4; we found it necessary to address research questions 2 & 3 in both rural-only and comparative thematic sections. The reason is based on methodological grounds, since the rural -only research is limited in generalizability; whereas the rural-urban comparative research has a benchmark against which we can contextualize the rural findings. We recognize that there is overlap exists among some of the themes. For example, seven studies were included in both the rural-only research and technology groups, while four studies were in both the comparative and technology groups. Table 5 also identifies the study design for each study, noting that the rural-only and technology groups were primarily qualitative, and the rural/urban comparative group was a mix of qualitative, quantitative and mixed-methods.

**Table 3:** Summary of themes and findings

Theme	Examples of Potential Factors/Mechanisms
Prevalence of Si/L	*Positive* – prevalence of Si/L, but lower than urban places. *Negative* – Urban areas were less lonely.
Rural focused research	*Positive* – the popularity of volunteering kept older rural residents connected to others during the pandemic. *Negative* – older adults who are caregivers are often more socially isolated than their peers, which leads to feelings of loneliness.
Rural/urban comparative studies	*Positive* – the close-knit social ties in rural places left older adults less socially isolated and lonely than those in urban centres. *Negative* – older adults in rural areas who have ceased driving found themselves more socially isolated and lonely during the height of the COVID-19 pandemic.
Technology and other interventions	*Positive* – rural dwelling older adults were quick to adapt to digital technologies to maintain contact with family and friends. *Negative* – the digital infrastructure was often seen to be lacking in rural places, which limited online connectivity.

**Table 4:** Methodological breakdown of included studies

Thematic Group	Breakdown (number of studies)		
	*Qualitative*	*Quantitative*	*Mixed Methods*
Prevalence of Si/L (*n* = 10)	0	6	4
Rural-only Research (*n* = 16)	12	2	2
Rural/Urban Comparative (*n* = 10)	4	4	2
Technological and other interventions (*n* = 14)	9	3	2

### Prevalence of social isolation/loneliness during the COVID-19 Pandemic (*n* = 10)

#### Positive outcomes for rural areas

Nine studies indicated that the prevalence of Si/L was less common in rural areas, compared to urban areas, with a primary focus on loneliness. These studies were conducted in five different countries, including Bangladesh [41, 42], Canada [43, 44], France [45], Serbia [46], the United States (US) [47], and the United Kingdom (UK) [48, 49]. Several studies determined rural areas to be a protective factor against Si/L, and identified women, those with financial challenges and additional chronic health issues, as groups with the highest prevalence or risk of loneliness [47–49]. Older rural residents in the US also had a lower prevalence of loneliness as compared to urban residents [47], and were much less worried about contracting COVID-19 [50]. The prevalence of loneliness was often connected to possible mental health issues, such as depression, and found to be similar to experiences of those in long-term care facilities [44, 46]. For example, Martin et al.’s (2022) study in rural Canada and found loneliness in 72% of respondents, with nearly 80% expressing concern for their mental health [44]. One study by Mistry et al. (2022a) conducted two periods of data collection in 2020 and 2021. In this study, the prevalence of loneliness was found to have dropped significantly from 2020 to 2021, which suggests some adaptation to the pandemic occurred in older, rural dwelling Bangladeshi adults [41].

#### Neutral outcomes for rural areas

Only two studies included for review found a neutral prevalence of loneliness in rural and urban places. These studies were conducted in Japan [51], and in the US [50]. Although limited in number, these studies suggest that rural areas are diverse and unique spaces with the potential to vary greatly. In the Japanese context, loneliness was prevalent, but rural or urban locations were found to be statistically insignificant when comparing levels of loneliness [51]. This study found that the prevalence of loneliness was quite high in rural and urban areas, even prior to the pandemic, and neighbourhood-based factors were likely the best protections against loneliness. Hennig-Smith et al. (2022b) found loneliness in the rural US to be prevalent in both urban and rural environments; however, the effect on mental health and social well-being outcomes for both rural and urban respondents was the same [50].

### Rural-only research (*n* = 16)

#### Positive outcomes for rural places

In five of the sixteen rural-focused studies (i.e., based solely on rural participants), older adults were shown to be highly engaged socially in their daily lives and rural-dwelling older adults were more likely to report that they were supported [11, 12, 51, 53]. In fact, Colibaba et al. (2021) noted that older adults continued their practice of voluntarism to stay connected to the community during the pandemic and found the rural environment to be more suited to the lockdowns [52]. This was echoed by other studies, which found that older adults relied on past experiences of isolation to manage their mental health during the pandemic [11, 12]. Rural residents could go outside, in their gardens for example, without the fear of being in close contact with others [53]. The mechanisms contributing to protections against loneliness were quite varied amongst the studies. The most common reported activity that helped older adults maintain social connection during the COVID-19 pandemic and lockdowns was volunteering [11, 12, 44, 52]. Volunteering acted to foster forms of support such as combating institutional changes (e.g., support for online learning and assistance with home schooling of children) and bridging gaps where existing infrastructure did not exist or was inadequate (e.g., the reallocation of transportation options when buses and taxis were not readily available) [11, 12, 52] found that the role of rural public libraries pivoted to create virtual meeting spaces for the local residents, including older adults [54].

#### Negative outcomes for rural places

Rural-only studies (*n* = 9) that found negative aspects linked to rural environments depicted a wide-range of environmental specific challenges, often for the most at-risk older adults. Older adults who were already experiencing Si/L prior to the pandemic and subsequent lockdowns became extremely isolated, and often lonely. This included those who were living alone [55], older adults with chronic health or substance abuse problems [44, 55], informal caregivers (i.e., often older adults providing spousal care) [43], and those who had recently moved to rural environments [56]. The lack of ability to connect with family, friends and routine social engagements caused significant mental health harm, often from a lack of rural-specific information and the fear surrounding COVID-19 infection and sickness [53, 57]. However, these challenges were identified in studies focused on populations in the *Global North* (i.e., more affluent, developed and powerful countries). 4 studies in the *Global South* (i.e., less affluent, underdeveloped countries) [58–61] exposed the precarious nature of ageing in rural places during the COVID-19 pandemic without official support infrastructure. In Nigeria, Ekoh et al. (2020, 2021, 2022) reported financial disaster for the rural-based older adults who depended entirely on familial support systems [58–60]. When these supports were disrupted by government mandated lockdowns, some older adults were left to starve with no way to travel to get food and no means to make purchases. In Ghana, similar depictions of the experience of older adults were made with a focus on those suffering from existing health issues, including blindness [61].

### Rural/urban Comparative Articles (*n* = 10)

#### Positive outcomes for rural places

This theme was chosen based on methodological grounds, since comparative studies provide a benchmark against which rural findings can be contextualized. Six studies comparing rural and urban experiences during the COVID-19 pandemic identified protective factors of the rural environment for the Si/L of older adults [27, 45, 47–49, 62]. Peres et al. (2021) found that older rural residents were three times more likely to feel supported compared to urban dwellers, and further, that urban residents reporting twice the level of negative social experiences compared to their rural counterparts [45]. Rural environs are often close-knit communities, where rural residents are well connected and supportive of one another, which may be indicative of greater resilience to adversity in the form of social isolation [47]. Older rural residents often have family living close-by and were more likely to have visitors during the pandemic than their urban counterparts [45, 47, 49]. In Japan, older customs that are no longer relevant or active in urban areas were revived to help older residents stay connected. The revisiting of old customary ‘neighbourhood conferences’ (*Osekkai)* greatly reduced feelings of loneliness and helped build social connections [62].

#### Negative outcomes for rural places

Specific geographic characteristics of the rural environment, as compared to urban centres, were implicated in four studies as exacerbating experiences of loneliness and contributing to the deterioration of mental health during the COVID-19 pandemic, particularly during lockdowns [42, 46, 56, 63]. Egeljić-Mihailović et al. (2022) found similar patterns of low social participation and depression symptoms in Serbia between those living in urban-based long-term care and those living in rural places [46]. Rural residents are heavily reliant on the few gathering spots available in their neighbourhoods and the loss of access to these places resulted in feelings of loss and loneliness [56]. Finlay et al. (2022) also found political differences in the US became more apparent when the social circles of older adults shrunk during the pandemic, particularly in rural areas [56]. The social challenges in rural contexts are further worsened by chronic health care issues and the lack of health care services as compared to urban environments [63]. Only one study included for review looked at the differences between urban and rural places and the experience of older adults during the COVID-19 pandemic. An urban/rural comparative study by Mistry and colleagues (2022b) found 53.4% of the rural-based participants to be lonely in 2020 compared to 43.7% of those in urban areas [42]. The key drivers of loneliness in this study were documented as financial strains and living alone in rural Bangladesh.

### Technological and other interventions (*n* = 14)

#### Positive outcomes for rural places

The digital divide differentiates people and communities in access and use of Internet technology [64], which has been applied to older adults living in rural places [65–67]. However, the majority of studies (*n* = 8) included in this review exploring the connection between ageing, rurality and technology during the pandemic found many rural-dwelling older adults were actually early adopters of technologies to maintain social and familial contact. The digital technologies were wide ranging, with some being institutional driven and others being individual driven. Lenstra and colleagues (2022) found that US public libraries pivoted to virtual services to help connect older adults in rural areas during the pandemic [54]; while Henning-Smith et al. (2022b) identified higher levels of social media use among rural dwelling older adults than those living in urban centres in the US [50]. The use of social media to replace face-to-face contact during the COVID-19 pandemic required significant effort on the older adults who chose to do so, including the creation of schedules and strategies to make it more effective [11]. When the digital infrastructure was sufficient, video chats (e.g., FaceTime, Skype) and social media messaging were the choice to stay connected, including the live-streaming of church services [56]. Lund and Ma (2021) found Facebook and Twitter to be the most common form of social media used by older adults in rural areas and small towns, and that older adults were most interested in news relating to health and politics [68]. Beyond the use of social media, other technologies were used to connect people, maintain social networks and stay mentally active. For example, a study conducted in Japan demonstrated that older adults were using video games on their phones as a way to connect to others, keep themselves mentally active, and to combat isolation and loneliness during the COVID-19 pandemic [53]. Even in cases where the infrastructure to support online digital technologies was lacking, older adults used cell phones and traditional landline phone calls to stay in touch with family and friends [11, 51, 59, 61].

#### Negative outcomes for rural places

Rural areas commonly experience varying degrees of digital poverty, as compared to urban places, where digital poverty refers to an inability to interact with the *online* world due to a lack of connectivity or technical ability [69]. Despite reports of general willingness and openness to using new digital technologies, such as social media platforms, older adults often identified these products as inadequate. Within the studies reporting negative experiences using technology, many rural residents were unsatisfied by digital methods to connect to friends and family and longed for face-to-face contact. In fact, Martin et al. (2022) reported older adults living in rural areas responded to the pandemic lockdowns by increasing their use of social media, but they still reported feeling lonely [44]. Many social media users were reporting on experiences in urban places, which caused rural residents to become fearful and confused, despite a relatively low risk of contracting COVID-19 in their immediate rural areas [62]. Additionally, the shift to using digital technologies was also cited as a barrier [52, 70]. Of those who chose not to use digital technology to combat Si/L, the most cited reasons included distrust of social media platforms [47, 70], lack of knowledge in terms of operation and digital skills [52], a lack on connectivity to their personal dwellings [71], and a lack of digital literacy and/or skills [52, 70].

**Table 5:** **Study Characteristics**

#	Author(s)	Article Title/Year	Design	Research	Notes/Comments
1	Bu, F., Steptoe, A., & Fancourt, D.	Loneliness during a strict lockdown: Trajectories and predictors during the COVID-19 pandemic in 38,217 United Kingdom Adults. (2020)	Quantitative	Comparative	Loneliness increased during the pandemic, but rural residency was seen as a protective factor. Living with others was also seen to be protective against loneliness.
2	Colibaba, A., Skinner, M. W., & Russell, E.	Rural Aging during COVID-19: A Case Study of Older Voluntarism. (2021)	Qualitative	Rural only and Technology	Volunteering was an important strategy to keep older adults connected during the pandemic. Digital based technology was seen to be effective when lockdowns limited face-to-face contact.
3	Diehl, C., Tavares, R., Abreu, T., Almeida, A.M.P., Silva, T.E., Santinha, G., Rocha, N.P., Seidel, K., MacLachlan, M., Silva, A.G. and Ribeiro, O.	Perceptions on Extending the Use of Technology after the COVID-19 Pandemic Resolves: A Qualitative Study with Older Adults. (2022)	Qualitative	Technology	Older adults, who are rural residents, were less interested to adopt new technology during the COVID-19 pandemic. The primary reasons for this were privacy concerns and a lack of digital skills amongst older adults.
4	Egeljić-Mihailović, N., Brkić-Jovanović, N., Krstić, T., Simin, D., & Milutinović, D.	Social participation and depressive symptoms among older adults during the Covid-19 pandemic in Serbia: A cross-sectional study. (2022)	Quantitative	Comparative	Older adults in urban areas scored higher on a depressive symptoms scale than those living in rural areas. Residents of rural areas in developed countries have higher depression rates.
5	Ekoh, P. C.; Agbawodikeizu, P. U.; Ejimkararonye, C.; George, E. O.; Ezulike, C. D.; Nnebe, I.	COVID-19 in rural Nigeria: Diminishing social support for older people in Nigeria. (2020)	Qualitative	Rural only	The COVID-19 pandemic has led to reduced support for rural based older adults, including food, financial, and social support (i.e., assistance, communication, care), due to limited social contact in Nigeria. adults in Nigeria.
6	Ekoh, P. C.; George, E. O.; Ezulike, C. D.	Digital and Physical Social Exclusion of Older People in Rural Nigeria in the Time of COVID-19. (2021)	Qualitative	Rural only and Technology	The digital divide affects rural areas in Nigeria, but the use of low-tech devices, such as the radio, was useful in reducing Si/L.
7	Ekoh, P. C., George, E. O., Agbawodikeizu, P. U., Ezulike, C. D., Okoye, U. O., & Nnebe, I.	“Further Distance and Silence among Kin”: Social Impact of COVID-19 on Older People in Rural Southeastern Nigeria. (2022)	Qualitative	Rural only	This study exposed dependence on familial and social networks for support in Nigeria during the COVID-19 pandemic.
8	Finlay, J. M., Meltzer, G., Cannon, M., & Kobayashi, L. C.	Aging in place during a pandemic: neighborhood engagement and environments since the COVID-19 pandemic onset. (2022)	Qualitative	Comparative and Technology	This study highlights resilience among rural-based older adults and their neighbors during the COVID-19 pandemic.
9	Fuller, Heather R.; Huseth-Zosel, Andrea	Older adults’loneliness in early COVID-19 social distancing: implications of rurality. (2021)	Mixed-methods	Comparative and Technology	Small-town living may be protective for older adults during the pandemic.
10	Galkin, K. T.	“The body becomes closed and squeezed up in a narrow frame”: loneliness and fears of isolation in the lives of older people in rural areas in Karelia during COVID-19. (2020)	Qualitative	Rural only	Participants in this study drew connections between their rural environment and a prison during the COVID-19 pandemic. This was primarily due to a lack of connectivity and the lockdown orders.
11	Gorenko, J. A., Moran, C., Flynn, M., Dobson, K., & Konnert, C.	Social isolation and psychological distress among older adults related to COVID-19: a narrative review of remotely-delivered interventions and recommendations. (2021)	Qualitative	Technology	Digital connections and technology uptake by older adults were limited in rural places as compared to urban places
12	Hanesaka, H.; Hirano, M.	Factors Associated with Loneliness in Rural Older Adults during the COVID-19 Pandemic: Focusing on Connection with Others. (2022)	Quantitative	Rural only	The COVID-19 pandemic made older adults more aware of their own behaviours, and of the behaviours of others.
13	Henning-Smith, C.	The Unique Impact of COVID-19 on Older Adults in Rural Areas. (2020)	Qualitative	Rural only	Rural older adults living near the poverty line face challenges in the USA.
14	Henning-Smith, C., Tuttle, M., Tanem, J., Jantzi, K., Kelly, E., & Florence, L. C.	Social isolation and safety issues among rural older adults living alone: perspectives of Meals on Wheels programs. (2022a)	Qualitative	Rural only	Rural experiences were mainly negative. Data was collected from rural ‘meals on wheels’ services.
15	Henning-Smith, C., Meltzer, G., Kobayashi, L. C., & Finlay, J. M.	Rural/urban differences in mental health and social well-being among older US adults in the early months of the COVID-19 pandemic. (2022b)	Quantitative	Comparative and Technology	Rural dwelling older adults were less worried about potential outcomes of COVID-19 and relied more on social media than those in urban areas.
16	Herron, R. V.; Newall, N. E. G.; Lawrence, B. C.; Ramsey, D.; Waddell, Candice M.; Dauphinais, J.	Conversations in Times of Isolation: Exploring Rural-Dwelling Older Adults’ Experiences of Isolation and Loneliness during the COVID-19 Pandemic in Manitoba, Canada. (2021)	Qualitative	Rural only and Technology	Past experiences of isolation in rural places, negotiating physically distanced visits, connecting with others, and “keeping busy”were identified as resources and strategies to mitigate isolation and loneliness.
17	Herron, R. V., Lawrence, B. C., Newall, N. E., Ramsey, D., Waddell-Henowitch, C. M., & Dauphinais, J.	Rural older adults’ resilience in the context of COVID-19. (2022)	Qualitative	Rural only	Rural dwelling older adults were found to be highly resilient to the negative effects of the pandemic due to their experiences of living in social isolation.
18	Jensen, L.; Monnat, S. M.; Green, J. J.; Hunter, Lori M.; Sliwinski, M. J.	Rural Population Health and Aging: Toward a Multilevel and Multidimensional Research Agenda for the 2020s. (2020)	Qualitative	Comparative	Rural family caregivers were found to be particularly vulnerable to mental stress, social isolation and loneliness due to the burden of caregiving.
19	L’Heureux, T., Parmar, J., Dobbs, B., Charles, L., Tian, P. G. J., Sacrey, L. A., & Anderson, S.	Rural family caregiving: A closer look at the impacts of health, care work, financial distress, and social loneliness on anxiety. (2022)	Mixed-methods	Rural only	Rural based older adults who identified as informal caregivers were more socially isolated and lonely than their peers.
20	Lenstra, N.; Oguz, F.; Winberry, J.; Wilson, L.S.	Supporting social connectedness of older adults during the COVID-19 pandemic: the role of small and rural public libraries. (2021)	Quantitative	Technology	Rural public libraries operate as unofficial senior centers and switched to using virtual technologies to keep older residents connected during the pandemic.
21	Lund, B.; Ma, J.	Exploring information seeking of rural older adults during the COVID-19 pandemic. (2022)	Qualitative	Rural only and Technology	Older adults were most concerned with health and political information during the pandemic in rural areas.
22	Martin, C., Szabo, A., & Champlin, C.	A Study exploring the impact of Covid-19 on the mental and physical health of older adults in a small rural community: What we learned. (2022)	Mixed-methods	Rural only and Technology	Underlying health issues were identified as the major factor contributing to increased isolation and loneliness during the pandemic.
23	Mistry, S. K., Ali, A. R. M. M., Yadav, U. N., Khanam, F., & Huda, M. N.	Changes in loneliness prevalence and its associated factors among Bangladeshi older adults during the COVID-19 pandemic. (2022a)	Quantitative	Comparative	Loneliness levels are high in rural-dwelling older adults, especially for those with poor finances and living alone. However, loneliness actually decreased from 2020 to 2021.
24	Mistry, S.K., Ali, A.M., Yadav, U.N., Huda, M.N., Ghimire, S., Saha, M., Sarwar, S. and Harris, M.F.	Loneliness and its correlates among Bangladeshi older adults during the COVID-19 pandemic. (2022b)	Quantitative	Comparative	The findings in this study suggest specific policies and plans are needed to reduce loneliness among older adults who require additional care in Bangladesh.
25	Ohta, R.; Yata, A.	The revitalization of “Osekkai”: How the COVID-19 pandemic has emphasized the importance of Japanese voluntary social work. (2021)	Qualitative	Comparative and Technology	Rural residents were forced to behave in the same way as urban residents and received most of their news from urban centres. This exacerbated challenges in rural places for older adults.
26	Pérès, K.; Ouvrard, C.; Koleck, M.; Rascle, N.; Dartigues, J-F.; Bergua, V.; Amieva, H.	Living in rural area: A protective factor for a negative experience of the lockdown and the COVID-19 crisis in the oldest old population? (2021)	Mixed-methods	Comparative	The rural environment was reported to have a protective effect in comparison to urban places. The COVID-19 virus arrived later in rural areas and people were both well informed and equipped to manage it.
27	Rutland-Lawes, J.; Wallinheimo, A-S.; Evans, Simon L.	Risk factors for depression during the COVID-19 pandemic: a longitudinal study in middle-aged and older adults. (2021)	Quantitative	Comparative	The close-knit social ties and sense of community in rural areas reduced loneliness for older adults during the COVID-19 pandemic., especially for older women.
28	Takashima, R.; Onishi, R.; Saeki, Ka.; Hirano, M.	Perception of COVID-19 restrictions on daily life among Japanese older adults: A qualitative focus group study. (2020)	Qualitative	Rural only and Technology	Rural dwellers were more worried about how they are viewed by neighbours. This even limited all forms of travel outside the home during the pandemic lockdowns.
29	Tsiboe, A. K.	Describing the experiences of older persons with visual impairments during COVID-19 in rural Ghana. (2020)	Qualitative	Rural only	Negative experiences of rural-based older adults in Ghana during the pandemic were exacerbated by underlying health conditions.

## Discussion

In this scoping review, we investigated the effects of social isolation and loneliness of older adults living in rural areas during the COVID-19 pandemic. Following an extensive review of the English-language scholarly literature, we analyzed twenty-nine qualitative, quantitative and mixed method articles in the existing literature. We summarized insights into the following four broad themes: (1) Prevalence of social isolation and loneliness, (2) Rural-only research, (3) Urban-Rural comparative research, and (4) Technological and other interventions (Table 3). These thematic categories allowed for a more structured analyses of the studies based on our research questions. The factors central to each of these themes, and related sub-themes, ultimately serve to influence older persons’ experiences during the COVID-19 pandemic, and the related lockdowns. We also acknowledge that there are interrelationships and a few contradictory findings among the themes, which are indicative of the need for further study in this area. For example, while digital technology and connectivity were found to be limited in some rural areas, other studies reported on the ability of older adults to adopt new technologies, such as social media, to better maintain their social connections. In addition, other factors such as socio-economic status (SES) likely have an effect on the ability of older adults to manage the pandemic and eventual lockdowns. Few articles address these topics and none of the included articles specifically examined aspects such as income, education, and literacy levels. SES would affect both the availability and affordability of technologies that are available in rural areas, creating a dichotomous experience between wealthier and poorer older adults.

The insights offered by these studies provide a wealth of information relevant to the current scoping review. From a human geographic perspective, the rural environment was more suited to the lockdowns because residents could go outside without the fear of being in close contact with others [e.g., 49, 53, 56]. In fact, the inherent experience of geographic isolation in rural places appears to have a positive effect on the resiliency of older adults in terms of their experiences of Si/L [e.g., 47]. Most included studies found the experience of living in isolation to be a protective factor against feeling lonely. The prevalence of loneliness was measured in ten of the thirteen studies relying on quantitative or mixed-methods approaches. Qualitative studies (*n* = 16) also described feelings of loneliness experienced by older adults living in rural areas during the pandemic. However, the included studies provided conflicting accounts of those in rural places. Studies conducted in the *Global South* [41, 42, 58–61] depicted the rural environment as an exacerbating factor for loneliness, those studies conducted in the *Global North* found the rural environment to be protective or insignificant. Finally, technology was an important tool used by older adults to minimise the effects of Si/L. Fourteen of the included articles specifically explored technology use, which was either high-tech (i.e., social media, video calls) or low-tech (i.e., phone calls, radio programs). The effectiveness of technology to impact Si/L was split with eight studies identifying a positive effect, and six identified a lack of effectiveness or uptake. However, the types of technological interventions are not available in all rural locations, and the included studies highlight these geographical variations and differences. Low-tech devices were the primary technological tools used in LMICs and less affluent areas, detailed by Ekoh et al. (2020, 2021, 2022), Mistry et al. (2022a, 2022b) and Tsiboe et al. (2020) [41, 42, 58–61]. Less distinction was made in more affluent countries of the *Global North*, such as Japan, where descriptions included somewhat ambiguous terms such as “social media” [e.g., 62] and “phone-based video games” [e.g., 53].

### Research gaps

A key aspect of scoping reviews is to help researchers identify knowledge gaps in the existing literature [35], and several were determined as a result of the current review. While all included studies addressed issues pertaining to Si/L, most studies focused on loneliness, not social isolation. Studies focusing on mental health outcomes, depression and the support systems older adults in rural areas rely on would add to the current discourse. It was also evident that studies focusing on countries in the *Global North* were over-represented as compared to studies focusing on the *Global South*. As a consequence, relatively little is known about the geographic differences of Si/L experiences of rural-based older adults. Additionally, rural spaces often lack a specific identity and are identified by what they are not, as opposed to what they are. For example, some included studies included small towns, whereas others explored the experiences in remote areas with very limited and disperse populations. Furthermore, the lack of clarity surrounding the definition of ‘rural’ creates a potential for ecological fallacy – is it an individual isolation experience that is accounting for the association; or, is it the context of the physical environment that accounts for isolation? The resources available in places are often highly varied, specifically in regards to broadband connection for internet and online services. The experiences of these older adults may be very different, despite the fact they might both be identified as rural residents. Researchers seeking to make advances in understanding these factors and experiences will need to identify novel ways of assessing rurality in ways that can be comparable among countries. Additionally, theoretical framing of rural aging may benefit from strength-based resilience and aging models that employ socio-environmental processes to address adversity, such as a pandemic, by integrating individual, community and system-level environmental domains [71].

Studies examining marginalised populations were lacking in this review. Few studies explored lived experiences relating to gender, sexual orientation, and racial or ethnic variations. It is important to highlight the relative lack of studies reporting on non-binary experiences during the lockdowns in general, and more specifically in rural locales. Research that examines such differences, as well as their intersections, can aid in achieving a more nuanced understanding of the factors synthesized, as shown in Table 4. Such insights would be valuable for policy-makers at the municipal, state/province/prefectural and national levels. Building on the abovementioned point, all research directions identified here can benefit from both quantitative and qualitative insights, and we encourage the use of a broader range of mixed methods and the identification of new sources of data. Future studies may benefit from adopting qualitative methods, other than interviews and surveys, such as using personal diaries, mapping exercises, or creating focus groups to expand our knowledge about the factors contributing to the Si/L among older adults in rural environments by drawing on more diversely-generated insights.

Finally, among the gaps in the literature, the lack of studies on LMICs was markedly important in this review. The negative experiences of Si/L resulting from living in rural places during the COVID-19 pandemic was clearer within LMICs. Rural residents are often naturally separated from their families [e.g., 59, 72] as many younger adults (i.e., children of rural-dwelling older adults) tend to migrate to urban centres for greater economic opportunities. The pandemic separated families, and many older rural residents were effectively cut off from their existing familial support structures. Although this was the case in many rural locations [50, 63, 73], the few studies on LMICs in the *Global South* (*n* = 6) provided a harrowing account of the experiences facing the most marginalised older adults [58–61]. Economic hardship was a reality before the lockdowns, but it was exacerbated during the pandemic [41, 42, 58–61]. The most vulnerable and least supported older adults living in rural areas of LMICs rely for support almost exclusively on their families, particularly on their adult children.

### Scoping review limitations

The main limitation to this study is that we omitted sources not written in English. Scoping reviews require parameters, and language is a commonly used one. We, thus, acknowledge that there may be robust scholarly discussions of related experiences in other languages that are not captured in the current review. Another limitation is that there is no universal definition of “*rural*.” As such, we acknowledge that our review will represent different conceptualisations of what defines rural or remote settings, particularly in different physical geographic locations and we may not have captured articles that described rurality using words other than those found in our keywords. We believe that this potential limitation was mitigated in part due to our post hoc review phase that involved hand-searching of the reference sections of fully reviewed sources, which is a hallmark of the Arksey and O’Malley (2005) scoping review protocol that we followed [35]. Further, while our thematic structure separated rural-only and rural-urban comparative studies, other approaches could be employed that are driven more by content-oriented themes. Finally, this “geropandemic” has been politically and culturally addressed disparately in different countries. This was apparent not only comparing the global south and north, but even occurring within the global north (e.g., Sweden vs. USA vs. Germany), and thus led also to different actions of shutdowns or restriction measures in different years (2020–2022). While we explored experiences relating to “lockdowns” imposed by the relative government authorities, it is important to recognise the differences among the various countries included in the reviewed studies.

## Conclusion

Many of the identified motivating factors of the Si/L of older adults living in rural environments during the COVID-19 pandemic seemed complex, interwoven and dichotomous. The juxtaposed nature in which the rural environment was characterised by studies in this review, positive and negative, highlights how little we know about ageing in rural and remote places. Much of the literature available focuses on countries in the *Global North* and cities. With rural places experiencing accelerated population ageing, as compared to urban centres, due primarily to younger people leaving for opportunities in cities [e.g., 74, 75], researchers need to further explore social isolation and loneliness in rural settings in earnest. Studies exploring the processes and outcomes of Si/L are currently lacking in the existing literature. The association between Si/L with various health issues and challenges underscores the need for more research. Expanding the variation of analytical approaches would also create interesting avenues for future research. This review highlights the lack of studies elucidating the interrelationships between contextual factors at both the macro and micro levels relating to the various processes and outcomes at the nexus of Si/L and ageing and place. Finally, the paucity of literature focusing on rural and/or rural/urban comparisons of Si/L, can be juxtaposed with a more prolific literature on urban dwelling older adults [e.g., 9, 24, 76–78]. Overall, this scoping review has systematically identified several important insights about existing knowledge of the experiences of older adults living in rural places during the COVID-19 pandemic, while also identifying the pressing knowledge gaps that can be addressed in future research.

## Data Availability

We have provided a detailed description of search strategies within this manuscript.

## List of Abbreviations

LTC: Long-term care
LMICs: Lower- and middle-income countries
SES: Social-Economic Status
Si/L: Social isolation and loneliness
UK: United Kingdom
US: United States
